# Obesity status trajectory groups among elementary school children

**DOI:** 10.1186/s12889-016-3159-x

**Published:** 2016-07-07

**Authors:** Tzu-An Chen, Tom Baranowski, Jennette P. Moreno, Teresia M. O’Connor, Sheryl O. Hughes, Janice Baranowski, Deborah Woehler, Rachel T. Kimbro, Craig A. Johnston

**Affiliations:** Center for Translational Injury Research, University of Texas Health Science Center, 6431 Fannin Street, Rm. 5.212, Houston, TX 77030 USA; USDA/ARS Children’s Nutrition Research Center, Baylor College of Medicine, Houston, Texas USA; The Oliver Foundation, Houston, Texas USA; Department of Sociology, Rice University, Houston, Texas USA; Department of Health & Human Performance, University of Houston, Houston, Texas USA

## Abstract

**Background:**

Little is known about patterns in the transition from healthy weight to overweight or obesity during the elementary school years. This study examined whether there were distinct body mass index (BMI) trajectory groups among elementary school children, and predictors of trajectory group membership.

**Methods:**

This is a secondary analysis of 1651 elementary school children with complete biannual longitudinal data from kindergarten to the beginning of 5^th^ grade. Heights and weights were measured by trained school nurses using standard procedures at the beginning and end of each school year for 11 consecutive assessments. Group-based trajectory clustering and multinomial logit modeling were conducted.

**Results:**

When using BMIz score, six trajectory groups were identified revealing substantial consistency in BMIz score across time. When using a categorical variable separating overweight/obese children (BMI ≥ 85%ile) from the rest, five developmental trajectories (persistently non-overweight/obese weight: 51.1 %; early-onset overweight/obese: 9.2 %; late-onset overweight/obese: 9.7 %; becoming healthy weight: 8.2 %; and chronically overweight/obese: 21.8 %) were identified. When using a categorical variable separating obese children (BMI ≥ 95%ile) from the rest, three trajectories (persistently non-obese: 74.1 %, becoming obese: 12.8 %; and chronically obese: 13.2 %) were identified. For both cutoffs (≥ BMI percentile 85 % or 95 %), girls were more likely than boys to be classified in the persistently non-overweight and/or obese group (odds ratios (OR) ranged from 0.53 to 0.67); and Hispanic children and non-Hispanic Black children were more likely to be chronically overweight and/or obese than non-Hispanic White children (OR ranged from 1.57 to 2.44). Hispanic children were also more likely to become obese (OR: 1.84) than non-Hispanic White children when ≥ BMI percentile 95 % was used.

**Conclusions:**

Boys, Hispanic and non-Hispanic Black children were at higher risk of being overweight or obese throughout their elementary school years, supporting the need for obesity treatment. Post kindergarten and post second grade summer months were times when some children transitioned into overweight/obesity. It will be important to identify which behavioral factors (e.g., diet, physical activity, sedentary behaviors, and/or sleep) predisposed children to becoming overweight/obese, and whether these factors differ by time (Kindergarten versus second grade). If behavioral predisposing factors could be identified early, targeted obesity prevention should be offered.

## Background

Obesity is a serious public health problem which adversely affects children’s health [1]. Childhood obesity is related to physical diseases, such as cardiovascular disease [2–4], type 2 diabetes [3, 5], some cancers [6], stroke [2], arthritis [6], sleep apnea [7], early adult mortality [8], and poorer mental health [9, 10], e.g. negative self-image [11] and peer perceptions [12].

Although the prevalence of childhood obesity may have leveled off among U.S. children in recent years [13, 14], it remains high: currently 34 % of school-aged children 6 to 11 years old were overweight or obese [13]. Children who were overweight or obese were more likely to be overweight or obese in adulthood [6, 15–18]. Little is known, however, about body mass index (BMI) developmental trajectories during childhood. Approximately 15 % were chronically obese from 9 to 16 years old, while 7 % became obese in adolescence [19]. A semi-parametric clustering procedure has categorized children using annual BMI into trajectory groups [20, 21]. Among Taiwanese elementary school children, four groups were identified with persistent relative weight status over time. Lin et al. [22], we applied this relatively new statistical procedure to semiannual elementary school children data in a community in the southwestern U.S.

Seasonal variation has been demonstrated in children’s BMI or standardized BMI z-score (BMIz): children’s overweight or obesity status increased during the summer months of the elementary school years [23]. Overweight and ethnic minority children gained more weight in the summer [24]. Weight status transition probabilities also differed by demographics, i.e., boys had higher probability of transitioning into the overweight/obese category than girls [25].

Gender differences have been reported in trends in children’s weight status [13, 26–28]. Ethnic disparities emerged at very young ages [29–31] and racial differences were found in longitudinal tracking patterns [24, 25, 32, 33]. Low SES groups disproportionately became obese [29, 34–36]. To our knowledge, no research has examined childhood BMI developmental trajectories among US elementary school children using semiannually collected data. This secondary analysis aimed to: 1) replicate in a US sample of elementary school children from 5 to 12 years old previous trajectories in BMIz scores over a five-year period; 2) identify the number and type of distinct categorical BMI percentile (overweight/obese versus others; obese versus others) trajectories; and 3) examine whether these trajectories were associated with gender, ethnicity, or socioeconomic status (SES). The identification of childhood weight status trajectory groups and their demographic correlates could inform obesity prevention interventions.

## Methods

### Participants

The original study included 3734 students enrolled in the 2005 kindergarten class from a Southeast Texas Independent School District (ISD), which included 45 elementary schools. The data were collected as part of the ISD’s administrative reporting responsibilities to the state of Texas and thereby obtained nearly complete population data for the kindergarten cohort. The administrative nature of the data set, alternatively, limited the variables available for analysis. Forty-one of 45 elementary schools in this ISD participated with over 80 % student participation in all schools. Baseline measurement data were collected on all children in the schools during data collection days (*n* = 3734). Not all children were available in school on subsequent data collection days. Some children left the school system (*n* = 899), while 1653 children had complete data at all 11 data assessments across 5 years. The ISD provided coded data which allowed children to be followed longitudinally. A full description of the sample, setting, and data collection methods can be found elsewhere, where seasonal differences were demonstrated in mean BMIz change [24, 32]. No significant differences were detected in gender or weight status between the children in these analyses (*n* = 1653) versus those not because of incomplete data (*n* = 2081). Significant differences were detected for ethnicity (*χ*^2^(4) = 108.24, *p* < 0.001) and school SES (*χ*^2^(1) = 156.74, *p* < 0.001): more non-Hispanic Black, Hispanic and low SES school children had missing data after the baseline measurement.

### Measures

School nurses measured children’s height and weight twice annually in the fall (end of August-beginning of October) and spring (end of March-beginning of May) of each school year. Tanita Accustat stadiometers were installed in all schools, which were also provided with a Tanita HD-351 Digital scale. Children wore light clothing without shoes, and height and weight were assessed at each time point during the longitudinal study. Details regarding training of the nurses have been described elsewhere [32]. The study protocol was approved by the school district and the Institutional Review Board of the Baylor College of Medicine.

Body mass index (BMI) was calculated using the standard formula (weight (lbs.)/[height(in)]^2^ × 703) and translated into standardized (BMIz) and percentile scores using gender and age normative data from the Centers for Disease Control and Prevention Growth Charts for the United States [37].

Based on BMI percentiles, children were classified into one of three weight categories using established definitions: healthy weight (<85^th^ percentile for BMI), overweight (≥85^th^ percentile and <95^th^ percentile for BMI), or obese (≥95^th^ percentile for BMI) at each assessment. Given the small number of children in the underweight category (<5^th^ percentile BMI) (*n* = 54), they were included in the healthy weight group for this analysis.

Compared with the nationally representative National Health and Nutrition Examination Survey (NHANES) data, this sample of elementary school aged children from a school district in the southwestern US had a prevalence of overweight or obesity (27.44 % to 38.04 %) similar to the nationally representative NHANES of 34 % of 6–11 year old children [13]. The percent of overweight or obese among younger children was somewhat higher (27.44 % to 28.53 %) than the 26 % (ages 5 and 6) and 27 % (age 7) in NHANES. The obesity rate in Texas has been reported to be higher than the national estimate [38].

The potential predictors of group trajectory included child’s gender, race/ethnicity, and SES. Race/ethnicity was based on parent report and was grouped into five categories: Hispanic, non-Hispanic black, non-Hispanic white, Asian/Pacific Islander, and Native American. Given their small number (*n* = 2), Native Americans were excluded from these analyses. School Title I status was used as a school level SES indicator as the researchers were not given access to records about whether students qualified for free or reduced price lunch. The elementary schools were identified as low SES campuses if receiving Title I funds; otherwise, the schools were treated as moderate to high SES campuses. Fifteen elementary schools were designated as Title I schools indicating 40 % or more of students were from low income families [39]. The final sample was 1651 children.

### Analyses

The 11 sequential BMIz measurements were fit into a semiparametric mixture, group-based trajectory model (GBTM) [40] using the SAS PROC TRAJ procedure [41] in SAS 9.4 [42]. GBTM was developed to identify the optimal number of distinct groups that classify individuals according to their longitudinal pattern. GBTM identified groupings of individuals incorporating all the data points instead of a single point of time.

The dependent variables were 1) BMIz, 2) presence or absence of overweight or obesity (≥85^th^ percentile for BMI), and 3) presence of absence of obesity (≥95^th^ percentile for BMI) at each data collection point. Three GBTMs were conducted, one for each dependent variable. The analysis on BMIz was conducted to assess correspondence in outcomes between a US sample with those reported elsewhere. Change in BMIz was the variable most highly correlated with age and sex-adjusted change in fat mass, and thereby most appropriate for these analyses. The analyses on the categorical variables were conducted to explore child movements over time above or below meaningful health related cutpoints [43]. GBTM requires the specification of the order of the polynomial equation for the trajectory [40]. Given 11 data points, the polynomial equation for the trajectory was specified as cubic:

$$ {Y}_{it}^{*j}={\beta}_0^j+{\beta}_1^j ag{e}_{it}+{\beta}_2^j ag{e}_{it}^2+{\beta}_3^j ag{e}_{it}^3+\varepsilon $$

where *Y*_it_^* *j*^ could be viewed as a latent variable measuring the behavior of interest (here, child’s BMIz or weight status), *age*_*it*_ is individual *i*’s age at time *t* given membership in group *j, ε* is a disturbance term assumed to be normally distributed with mean of zero and constant variance *σ*^2^, and *β*_1_^*j*^, *β*_2_^*j*^, *β*_3_^*j*^ are the parameters of the pattern of trajectory.

To evaluate the precision of trajectory group assignment, the metric of posterior group membership probability, i.e., the probability of each individual belonging to each trajectory group [44], was provided. Each child was classified into one trajectory group to which he/she had the highest posterior group membership probability of belonging. For example, a child who was chronically obese across the 11 time points had an estimated posterior probability of this child’s belonging to a persistently non-obese trajectory group close to zero, whereas the posterior probability estimate of the child’s belonging to a persistently overweight/obese trajectory group would be high. The estimated group probability was based upon the maximum likelihood of the trajectory parameters. The maximum likelihood aggregated the *J* likelihoods, *P*^*j*^(*Y*_*i*_) to form the probability of the data, *Y*_*i*_*:*

$$ P\left({Y}_i\right)={\displaystyle {\sum}_j{\pi}_j{P}^j\left({Y}_i\right)} $$

where *P*^*j*^(*Y*_*i*_) denotes the probability of *Y*_*i*_ given membership in group *j*; *π*_*j*_ represents the probability of membership in group _*j*_; and *P*(*Y*_*i*_) is the probability of observing individual *i*’s longitudinal sequence of outcome measurements.

Procedures described by Nagin [40] were followed to select the best fitting models. A series of models with progressively more trajectory groups were computed. Comparative model fit was evaluated using 1) Bayesian Information Criterion (BIC) [45]: 2*ΔBIC* > 10 [22]; 2) a posterior probability of greater than 0.7 [40]; 3) trajectory group size of at least 5 % of the sample [22]; and 4) identified trajectories were distinct and interpretable [22]. BIC approximates Bayes factor which measures the odds of two competing models being the correct model. The BIC is always negative; therefore, a smaller absolute value of BIC (the lesser negative) indicates increasingly better-fitting models. The BIC criterion favors parsimonious models having fewer trajectory groups [46].

Two additional analyses were conducted once children were assigned to a BMI trajectory group. First, a chi-square test was conducted to assess whether individual demographic characteristics differed among BMI trajectory groups for both the GBTM with the dependent variable of BMI 85^th^ percentile (%ile) and BMI 95 %ile. Second, multinomial logit models examined the capacity of the potential predictors (i.e., gender, ethnicity, and school SES) to distinguish children in the BMI trajectory groups from the reference group of persistently non-overweight/-obese children, controlling for the levels of the other demographics. The BMI developmental trajectory groups were the dependent variable. The analyses were done separately for both the GBTM with the dependent variables of greater than BMI 85%ile and BMI 95 %ile.

## Results

### Baseline demographics

Of the 1651 children included in the analyses, 50.0 % were boys, 31.6 % were non-Hispanic White, 22.7 % were non-Hispanic Black, 23.0 % were Hispanic; 27.2 % of the children were from Title I schools (low SES school); and 69.3 % were initially healthy weight (Table 1).

**Table 1:** Sample characteristics

Characteristics	Number	Percent
Gender		
Male	826	50.03
Female	825	49.97
Ethnicity		
Non-Hispanic White	522	31.62
Non-Hispanic Black	375	22.71
Hispanic	379	22.96
Asian	375	22.71
Initial Weight Status		
Underweight	54	3.27
Healthy Weight	1144	69.29
Overweight	238	14.42
Obese	215	13.02
School Title I (SES)^a^		
High SES	1202	72.8
Low SES	449	27.2

*Note.* ^a^Title I status indicates that at least 40 % of students come from low-income families

**Fig. 1:**
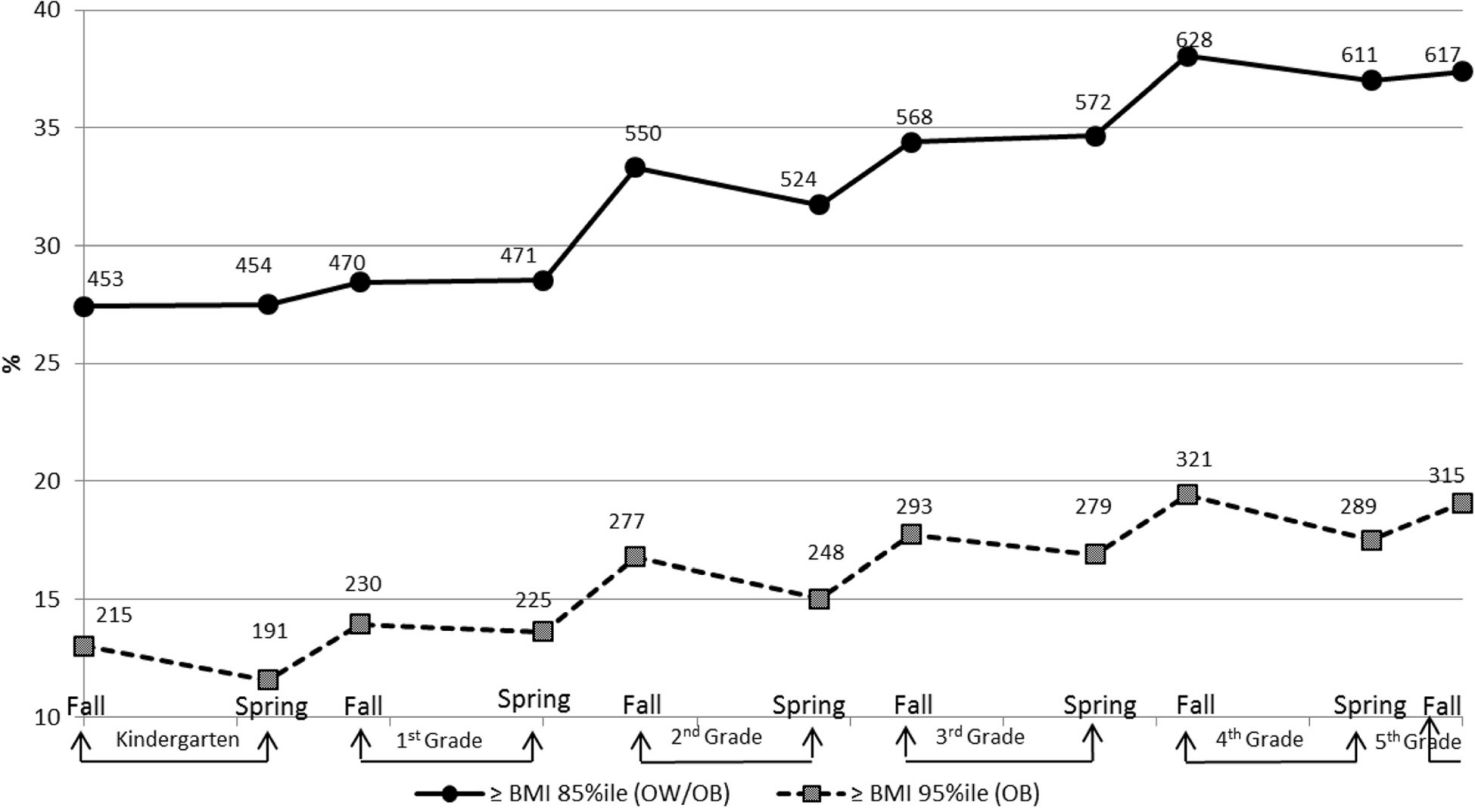
Percentages of Children Overweight and/or Obese over Time. Note. OW/OB: Overweight/Obese; OB: Obese; The numbers in the graph identify the number out of 1651 total children represented by that data point

Figure 1 presents the overall prevalence of overweight or obesity, and obesity across time using the CDC criteria of age- and gender-specific BMI equal to or greater than 85^th^ percentile and 95^th^ percentiles. For both the BMI 85 %ile and BMI 95 %ile, the prevalence of overweight and/or obesity generally increased with age. Overweight and/or obesity prevalence rose during each of the summer months, and decreased or flattened during school years.

### Developmental trajectories

#### BMIz

**Fig. 2:**
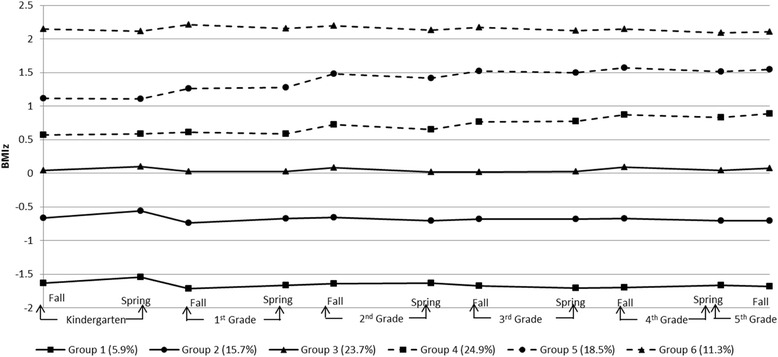
Trajectories of BMIz

All models had the average posterior probability greater than 0.7, and the trajectory group sizes were all greater than 5 % (data not shown). Based on the BIC tests, the six-trajectory model best fit the data (BIC: -12828.77). The dominant pattern was one of stability in BMIz, with increases over the 5+ years occurring only in the 2^nd^ and 3^rd^ highest BMIz groups (Fig. 2). Slight seasonal differences in BMIz were shown with summertime increases occurring primarily in the three higher BMIz trajectories (Fig. 2).

#### Overweight/obese: ≥ BMI 85%ile

When the dependent variable of the GBTM was BMI *≥* 85 %ile, improvements in BIC were obtained as the number of trajectory groups increased from two to six (BIC: -5874.88 ~ -4769.61). A five-trajectory model was chosen based on the model having converged, the largest BIC, 2*ΔBIC* > 10, posterior probabilities >0.7, each trajectory group size was greater than 5 % of the sample, and identified trajectories were interpretable (Table 2).

**Table 2:** BIC and Posterior Probability for Different Numbers of Groups of Trajectories by Different BMI %ile Criterion

Criterion	OW/OB (BMI ≥ 85 %ile)					OB (BMI ≥ 95 %ile)		
No. of trajectory groups	2	3	4	5	6	2	3	4
BIC								
All (*n* = 1651)	-5874.88	-5213.91	-4940.67	-4831.01	-4769.61	-3546.15	-3210.64	-3099.03
Average Posterior Probability								
All (*n* = 1651)	0.99	0.98	0.98	0.97	0.96	0.99	0.99	0.98

*Note*. BIC = Bayesian information criterion

**Fig. 3:**
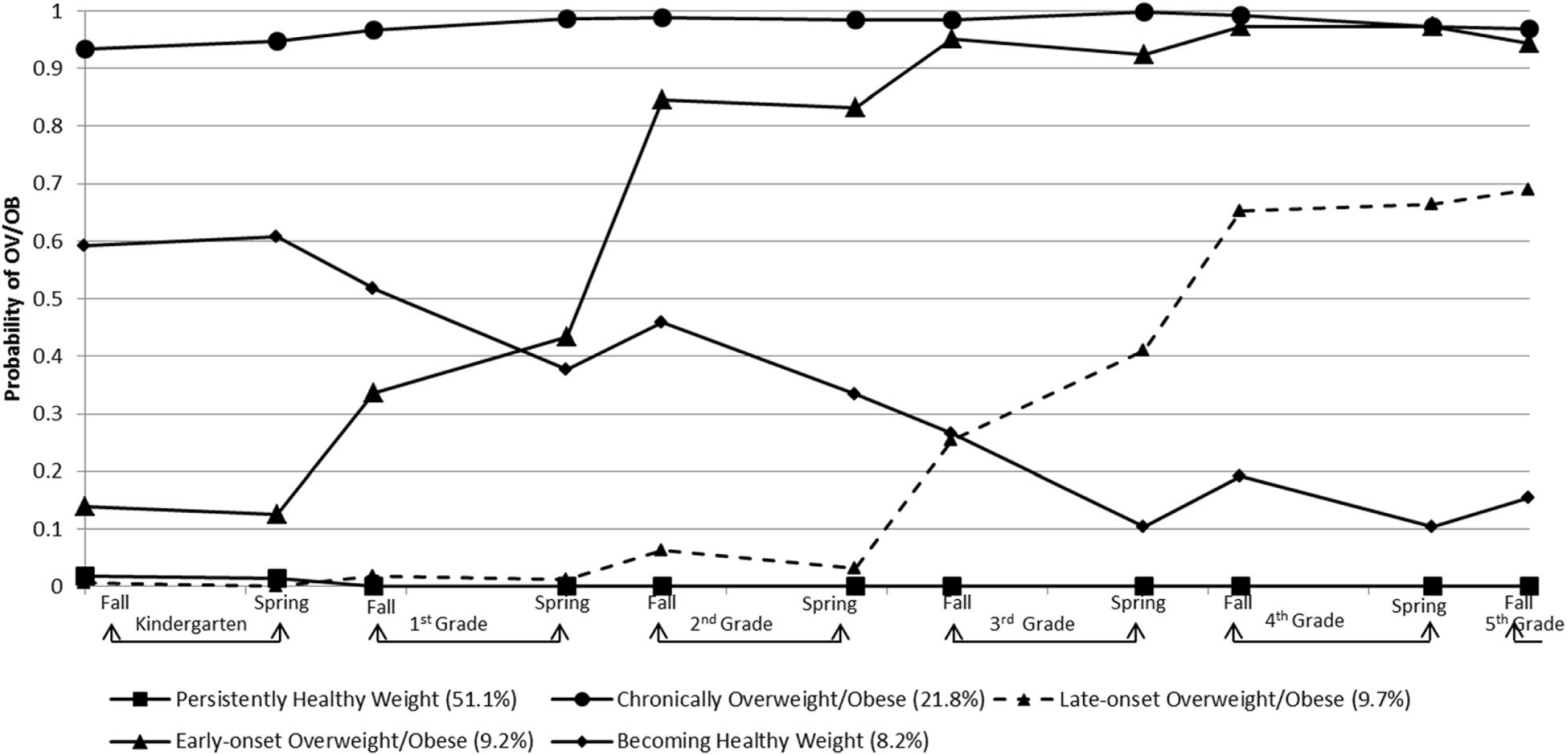
Trajectories of Overweight/Obese (BMI 85 %ile)

The five trajectory groups identified using the dependent variable of overweight/obese (BMI *≥* 85%ile) are shown in Fig. 3. This illustrates each child assigned to his/her most likely overweight/obese status developmental trajectory group based upon posterior probabilities. The lines shown in Fig. 3 were actual trajectories computed as the average BMI %ile of all the children assigned to the different groups identified by the best fitting model. The majority of children (51.1 %) were in the trajectory group named “persistently non-overweight/obese” and had very low probability of being in the overweight/obese category across the entire 11 time points. The second largest trajectory group was the “chronically overweight/obese” group (21.8 %) which remained at a high risk of being overweight/obese across all 11 observations. About 9.7 % of the children were classified in the trajectory group named “late onset overweight/obese”, who started in healthy weight range from Kindergarten through 2^nd^ Grade Spring, with their overweight or obesity risk increasing in the summer after 2^nd^ Grade through all of 4^th^ Grade. The “early onset overweight/obese” trajectory group included 9.2 % of the children, who had low obesity risk in Kindergarten, but tended to increase their probability of overweight/obesity in the summer after Kindergarten. Lastly, 8.2 % were classified into the trajectory group “becoming healthy weight”. These children were more likely to be overweight or obese at the beginning of Kindergarten, but their obesity risk decreased over time.

#### Obese: ≥ BMI 95%ile

For the GBTM with the dependent variable of BMI *≥* 95%ile, the ensuing analyses assessed multiple projectile groups of GBTM with each child assigned to his/her most likely obesity status developmental trajectory group based upon posterior probabilities. A six-trajectory model did not converge, but improvements in BIC generally were obtained as the number of trajectory groups increased from two to five (BIC: -3546.15 ~ -3055.28). However, the four-, five-, and six-trajectory models yielded at least one trajectory group size smaller than 5 % of the sample, thereby supporting a three-trajectory model.

**Fig. 4:**
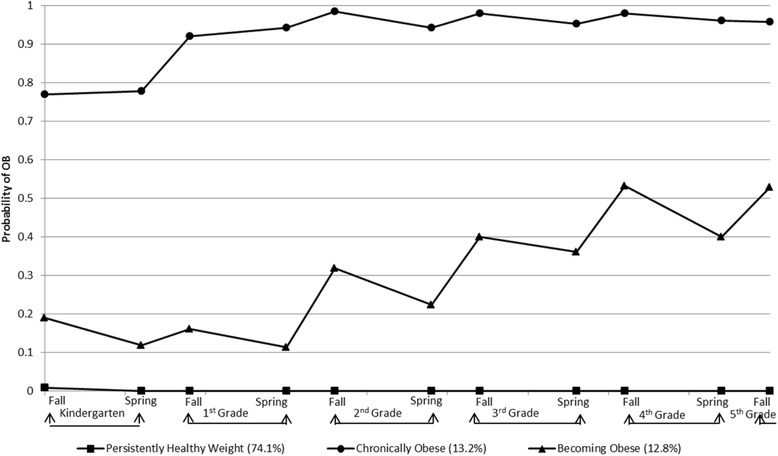
Trajectories of Obese (BMI 95 %ile)

Figure 4 shows the trajectories of estimated obesity status of the three-trajectory obesity status model. The trajectory group with the majority of the children (74.1 %), named “persistently non-obese”, had very low probability of being in the obese category across all measurement occasions. About 13.2 % of the children were classified in the trajectory group, “chronically obese”, showing a high probability of being obese at all the 11 time points. The smallest trajectory group, “becoming obese”, included 12.8 % of the children, who had an increasing probability of obesity over time. Figure 3 shows that the probability of obesity among the “becoming obese” group increased during most of the summer months compared to the school year.

### Demographics by overweight/obese trajectory groups

#### Overweight/obese: ≥ BMI 85%ile

Table 3 summarizes tests of bivariate relationships between the demographic characteristics with the trajectory groups in the overweight/obesity GBTM (dependent variable BMI *≥* 85%ile). Gender (*χ*^2^(4) = 25.85, *p* < 0.01), ethnicity (*χ*^2^(12) = 55.23, *p* < 0.01), and Title I School status (marker of SES) (*χ*^2^(4) = 27.92, *p* < 0.01) were all significantly different among the weight status developmental trajectories. Relatively fewer boys than girls were classified in the persistently non-overweight/obese group and more boys than girls classified in the chronically overweight/obese group. There were relatively more children from high SES schools, compared with children from low SES schools, in the persistently non-overweight/obese group than in the chronically overweight/obese group. Hispanic children were least likely to be in the persistently non-overweight/obese group and most likely to be in the chronically overweight/obese group followed by non-Hispanic Black children, non-Hispanic White children, and Asian children, showing increasingly disparate membership between groups.

**Table 3:** Participants’ Characteristics by Obesity Trajectory Groups with BMI 85%ile Criterion

	Persistently Non-Overweight/Obese		Late-onset Overweight/Obese		Early-onset Overweight/Obese		Becoming Healthy		Chronically Overweight/Obese	
	n	%	n	%	n	%	n	%	n	%
Gender***										
Male	375	45.4	88	10.65	78	9.44	80	9.69	205	24.82
Female	476	57.7	73	8.85	65	7.88	55	6.67	156	18.91
Ethnicity***										
Non-Hispanic White	292	55.94	50	9.58	43	8.24	48	9.2	89	17.05
Non-Hispanic Black	184	49.07	40	10.67	27	7.2	31	8.27	93	24.8
Hispanic	151	39.84	35	9.23	40	10.55	31	8.18	122	32.19
Asian	224	59.73	36	9.6	33	8.8	25	6.67	57	15.2
School Title I^a^ (SES)***										
High SES	658	54.74	117	9.73	99	8.24	101	8.4	227	18.89
Low SES	193	42.98	44	9.8	44	9.8	34	7.57	134	29.84

*Note.* Chi-squared test of independence was used to assess significance; ***p < 0.001; Percentages are calculated for each row; ^a^Title I status indicates that at least 40 % of students come from low-income families

Multinomial regression taking all variables into account simultaneously revealed that girls were less likely to be classified as late onset overweight/obese (OR = 0.63, 95 % CI: 0.45, 0.89), early onset overweight/obese (OR = 0.64, 95 % CI: 0.45, 0.92), becoming healthy (OR = 0.53, 95 % CI: 0.36, 0.76), or chronically overweight/obese (OR = 0.56, 95 % CI: 0.43, 0.72) than a persistently non-overweight/obese trajectory (Table 4). Compared to non-Hispanic White children, non-Hispanic Black children and Hispanic children increased in the odds ratio of group membership in chronically being overweight/obese by a factor of 1.57 (95 % CI: 1.08, 2.29), and 2.44 (95 % CI: 1.67, 3.58), respectively. School SES was not a significant predictor in the multivariate analyses.

**Table 4:** Comparison with Persistently Non-Overweight/Obese Weight Trajectory (BMI 85%ile criterion): Multinomial Regression

		Late-onset Overweight/Obese^a^		Early-onset Overweight/Obese^a^		Becoming Healthy^a^		Chronically Overweight/Obese^a^	
		Odds Ratio	95 % CI	Odds Ratio	95 % CI	Odds Ratio	95 % CI	Odds Ratio	95 % CI
Gender (reference group: Boy)	Girl	0.634**	0.451, 0.892	0.64*	0.447, 0.915	0.525***	0.362, 0.761	0.556***	0.431, 0.718
Ethnicity (reference group: White)	Asian	0.942	0.592, 1.497	1	0.615, 1.628	0.684	0.408, 1.145	0.836	0.573, 1.22
	Non-Hispanic Black	1.289	0.786, 2.114	0.933	0.534, 1.632	1.126	0.664, 1.911	1.574*	1.082, 2.291
	Hispanic	1.36	0.799, 2.315	1.636	0.952, 2.811	1.377	0.792, 2.394	2.444***	1.669, 3.577
School SES (reference group: High SES)	Low SES	1.081	0.69, 1.694	1.298	0.812, 2.072	0.934	0.571, 1.527	1.298	0.945, 1.783

*Note.* ^a^Reference group is persistently non-overweight/obese group; *p < 0.05; **p < 0.01; ***p < 0.001; CI: confidence interval

#### Obese: ≥ BMI 95 %ile

Table 5 shows the prevalence of demographic characteristics in each obesity trajectory group (BMI 95 %ile as the cut-point). Gender (*χ*^2^(2) = 15.66, *p* < 0.01), ethnicity (*χ*^2^(6) = 53.69, *p* < 0.01), and school SES (*χ*^2^(2) = 31.04, *p* < 0.01) were all significantly different among the weight status developmental trajectory groups. Girls were more likely to be classified as persistently non- obese, while relatively more boys were in the becoming obese and chronically obese groups. Hispanic children were least likely to belong to the persistently non- obese group and most likely to be in the chronically obese group. Relatively more children from high SES schools than children from low SES schools were in the persistently non- obese group while children from low SES schools were more likely to be in becoming obese and chronically obese groups.

**Table 5:** Participants’ Characteristics by Obesity Trajectory Groups with BMI 95 %ile Criterion

	Persistently Non-Obese		Becoming Obese		Chronically Obese	
	n	%	n	%	n	%
Gender***						
Male	580	70.22	118	14.29	128	15.5
Female	648	78.55	92	11.15	85	10.3
Ethnicity***						
Non-Hispanic White	414	79.31	58	11.11	50	9.58
Non-Hispanic Black	273	72.8	42	11.2	60	16
Hispanic	235	62.01	69	18.21	75	19.79
Asian	306	81.6	41	10.93	28	7.47
School Title I^a^ (SES)***						
High SES	937	77.95	137	11.4	128	10.65
Low SES	291	64.81	73	16.26	85	18.93

*Note.* Chi-squared test of independence was used to assess significance; ***p < 0.001; Percentages are calculated for each row; ^a^Title I status indicates that at least 40 % of students come from low-income families

As shown in Table 6, girls were less likely to be classified as becoming obese (OR = 0.67, 95 % CI: 0.50, 0.91), and staying obese (OR = 0.55, 95 % CI: 0.41, 0.74) than a persistently non- obese trajectory. Hispanic children were more likely to be classified as becoming obese (OR = 1.84, 95 % CI: 1.19, 2.86). Non-Hispanic Black children and Hispanic children had a higher odds ratio for group membership in the chronically obese group by a factor of 1.69 (95 % CI: 1.09, 2.62), and 2.37 (95 % CI: 1.52, 3.69), respectively. School SES was not a significant predictor in the multivariate analyses.

**Table 6:** Comparison with Persistently Non-Obese Weight Trajectory (BMI 95%ile criterion): Multinomial Regression

		Becoming Obese^a^		Chronically Obese^a^	
		Odds Ratio	95 % CI	Odds Ratio	95 % CI
Gender (reference group: Boy)	Girl	0.671**	0.498, 0.905	0.55***	0.407, 0.744
Ethnicity (reference group: White)	Asian	0.956	0.624, 1.466	0.76	0.467, 1.237
	Non-Hispanic Black	0.999	0.63, 1.585	1.688*	1.087, 2.624
	Hispanic	1.844**	1.189, 2.862	2.373***	1.524, 3.694
School SES (reference group: High SES)	Low SES	1.364	0.937, 1.986	1.362	0.952, 1.95

*Note.* ^a^Reference group is persistently non-obese group; *p < 0.05; **p < 0.01; ***p < 0.001; CI: confidence interval

## Discussion

This secondary analysis identified distinctive weight status trajectory groups for children by using GBTM to identify groups of children with different probabilities BMIz score and of overweight or obesity across the 5 years of assessments during elementary school.

Use of BMIz has been preferred over BMI percentile in longitudinal population-based analyses [43, 47]. Six stable trajectories were found when assessing BMIz over 11 time points. In contrast to our findings, among similar aged children from Canada [48] a four trajectory solution was determined with increasing BMIz for the lower three trajectories and stability only for the highest trajectory. Seasonal differences were not possible to detect in their biannual and annual data. Their high stable trajectory reflected higher child overeating, more mothers smoking during pregnancy, and more rapid weight gain in infancy. These variables were not available in our data set, but need to be assessed in future research.

In our GBTM model assessing the probability trajectories of overweight and obesity (BMI *≥* 85%ile), five overweight/obesity status trajectories were identified: 1) 51 % of the children who had a near zero probability of being overweight/obese at any time point, 2) 22 % who had a very high probability of being overweight/obese across all five years, 3) 10 % who increased their probability of overweight/obese starting at the summer after second grade, 4) 9 % transitioned their probability of overweight/obese in the early elementary school, and 5) 8 % who decreased their probability of overweight/obese (e.g. transitioned into healthy weight status). In the GBTM model assessing the probability trajectories of obesity (BMI *≥* 95%ile), three-quarters of children were classified as having a near zero probability of being obese at any time point (e.g. persistently non-obese weight) and 13 % made up each of the remaining two trajectory groups.

With the GBTM model for overweight or obesity (BMI *≥* 85%ile), the finding of five trajectory groups was different from the previously identified four obesity trajectories (boys: normal or slightly underweight, persistently normal weight, overweight becoming obese, and persistently obese; girls: persistently obese, persistently overweight, persistently normal weight, and persistently slightly underweight [22]) or three obesity trajectories for both boys and girls (gradual onset of overweight/at risk of overweight, always overweight/at risk of overweight, and normal weight [49]). Furthermore, our results identified two weight status trajectory groups which differed on when the children’s probability of overweight/obesity transitioned (starting in the summer right after Kindergarten or another right after 2^nd^ Grade). Some events (e.g., becoming healthy) were sufficiently rare that were not captured by the semiparametric mixture models in previous studies. Since the other study used data collected annually or biennially, it is possible that our larger sample or the more frequent semiannual longitudinal data enabled us to detect more nuanced trajectories. With the GBTM model for obesity (BMI *≥* 95%ile), one previous study also identified a three-trajectory group model, but different trajectory patterns (early onset overweight, late onset overweight, and never overweight [20]) using a different modeling approach (i.e., latent growth mixture modeling). The three trajectory groups (persistently healthy weight, becoming obese, and chronically obese groups) in this study were also inconsistent with two other prior findings identifying four-trajectory group models (no obesity, chronic obesity, childhood obesity, and adolescent obesity [19]; chronically obese, decreasing, increasing, and non-obese [21]). It is not clear if the number of data points, method of analysis, characteristics of sample population, or some other factor, accounted for these differences.

Previous studies reported seasonal differences in child’s weight or BMI [23–25, 50–55]. Significant differences in BMIz score in our study were found between the school year and summer months, but the pattern varied by trajectory group, with decreasing probability of increasing BMIz during summer months than in the school year in the lowest two trajectory groups for the first summer between Kindergarten and first grade. Future research will need to replicate these trajectories and assess seasonal differences in obesogenic behaviors and family and other influences on those obesogenic behaviors.

Inconsistent findings have been reported on gender differences in weight status trajectory groups. When assessing the probability of being overweight or obese (BMI *≥* 85%ile), one study showed that gender differed by obesity trajectory [21, 22]; however, another study indicated no gender difference [56]. The present study revealed that girls were less likely to be in the early onset overweight/obese, late onset overweight/obese, or becoming healthy trajectory groups, similar to a prior finding which utilized a slightly different developmental range (ages 7–12) [19, 21, 22]. In the models assessing the probability of obesity (BMI *≥* 95%ile) the finding in this study was in line with past research for adolescents (ages 6–18) [21, 22], indicating that boys were more likely to be chronically obese or becoming obese during both elementary schools and adolescence. Ethnic and school SES differences by overweight/obese or obese trajectory groups were consistent with past research. When BMI 95%ile was the cut-point, non-Hispanic White children were less likely to be chronically or becoming obese than non-Hispanic Black and Hispanic [21]. Ethnic differences among overweight/obese trajectory groups (BMI 85%ile cut-off) were not examined in previous studies.

Bivariate chi-square analysis revealed that overweight/obese or obese trajectory significantly varied among SES groups, which was consistent with previous findings, indicating an inverse relationship between the probability of becoming or remaining overweight/obese and SES [19, 57]. However, the effect was no longer significant after adjusting for other demographics. This was in contrast to that of previous studies [19, 22]. For BMI 85%ile cut-point, high SES (higher income or higher education level) was less likely to follow a becoming obese trajectory group than a persistently healthy weight trajectory group [21, 22]. For BMI 95%ile cut-point, children from low SES families were more likely to be in the chronic obesity or childhood obesity trajectory groups [19].

We previously applied different statistical procedures to analyze these data: mean change in BMIz over time [24], and Markov category transition probabilities over time [25]. Each procedure provided different insights into the dynamics of BMI change. The current procedure may provide the most interesting results for targeting interventions. Children who were chronically overweight and/or obese likely require an obesity treatment intervention while children who have a high probability of becoming overweight/obese at earlier (9.2 %) or later (9.7 %) points in elementary school may benefit from an obesity prevention intervention if they can be identified before or early in their transition. More research is needed to identify what factors (e.g., diet, physical activity, sedentariness, sleep) may account for who belongs in these groups, and how soon the differences emerge, so that interventions can be targeted to what may be expected to have the most preventive effect.

A strength of the current study was the use of objective measures with a large sample over five years, and the use of GBTM analysis. GBTM is conceptually similar to *K*-means cluster analysis, but has several advantages including measurement invariance [58], no prior specification of the number of groups to be extracted, and rigid model selection criteria. Alternatively, GBTM assumes zero within-class variances implying the trajectories of the individual-level group members are not allowed to vary about the group’s mean trajectory and the individual level heterogeneity can be expressed in terms of group differences [40, 59], and does not take into account cluster sampling [60]. Compared to several national surveys, the current sample size is relatively small; however, sample sizes of 500 can yield good estimates in the use of GBTM [61]. Limitations of the present study include an a priori assumption of the existence of distinct trajectories. Over- and under- fitting models were identified, implying the identified trajectories may merely reflect random variation, but unusual patterns were not identified. Trajectory assignments were based on probability of BMI %ile status category and are not absolutes [40, 59]. The findings are limited by the attrition and missing data which are relatively common in longitudinal studies. In addition, the sample was restricted to one school district in southeast Texas in the United States. For example, the summer changes may have been due to unusual heat or severe weather. Therefore, our findings may not be generalizable to the population of U.S. elementary school aged children, but the prevalence of obesity was similar.

## Conclusion

Six BMIz, five overweight/obese and three obese trajectory groups were identified. Subgroups changed status from kindergarten to the start of 5^th^ grade for the BMI 85^th^ percentile and the BMI 95^th^ percentile, respectively. The identification of possible factors influencing each subgroup would be important for the design of childhood obesity interventions to more precisely target summer behaviors and out-of-school influences that support these behaviors.

## Abbreviations

%ile, percentile; BIC, Bayesian Information Criterion; BMI, body mass index; CI, confidence interval; GBTM, group-based trajectory model; ISD, Independent School District; NHANES, National Health and Nutrition Examination Survey; OR, odds ratio; SES, socioeconomic status
